# Relationship between depressive symptoms and self-reported menstrual irregularities during adolescence: evidence from UDAYA, 2016

**DOI:** 10.1186/s12889-022-13196-8

**Published:** 2022-04-14

**Authors:** Priya Maurya, Trupti Meher, T. Muhammad

**Affiliations:** International Institute for Population Sciences, Mumbai, Maharashtra India 400088

**Keywords:** Depressive symptoms, Menstrual irregularities, Adolescents, India

## Abstract

**Background:**

The study examined the prevalence of self-reported menstrual irregularities during adolescence and explored the association of depressive symptoms with self-reported menstrual irregularities in adolescents in two major states of Uttar Pradesh and Bihar in India.

**Methods:**

This study is based on the data obtained from the first round of the "Understanding the lives of adolescents and young adults" (UDAYA, 2016) survey. The effective sample size for the study was 12,707 adolescent girls aged 10–19 years. A bivariate analysis with chi-square test was conducted to determine the self-reported menstrual irregularity by predictor variables. Multivariable logistic regression models were employed to examine the associations between self-reported menstrual irregularity, depressive symptoms and other explanatory variables.

**Results:**

A proportion of 11.22% of adolescent girls reported menstrual irregularity and 11.40% of the participants had mild depressive symptoms. Adolescent girls with mild (AOR: 2.15, CI: 1.85–2.51), moderate (AOR: 2.64, CI: 2.03–3.42) and severe depressive symptoms (AOR: 2.99, CI: 2.19–4.10) were more likely to have menstrual irregularity as compared to those who had minimal depressive symptoms. Physically active adolescent girls were less likely to report menstrual irregularity (AOR: 0.82, CI: 0.73–0.93) than physically inactive girls. Adolescent girls who used piece of cloth for menstrual hygiene practices (AOR: 1.17; CI: 1.02–1.35) and those who used either napkin or cloth or other materials (AOR: 1.32; CI: 1.14–1.54) had higher likelihood of menstrual irregularity as compared to those who used only sanitary napkins.

**Conclusion:**

A significant association of depressive symptoms with self-reported menstrual irregularity among adolescent girls was observed. Therefore, while treating females with irregular menstrual cycles, clinicians may need to pay greater attention to thir mental health peoblems.

## Background

Menstruation is one of the important changes that occur in adolescence as an indicator of women’s reproductive health and endocrine functions. Generally, regular menstrual cycle in females in their early ages occurs every 28 to 35 days with a variation of 2–3 days, in which the menstrual flow lasts for three to five days with an average loss of 30–80 ml of blood [1]. Epidemiologic studies have estimated that around 5% to 35% of women of reproductive age may experience some symptoms of irregular menstruation [2–4]. Out of this, 80% of the irregularity is attributed to pre-menstrual phase of menstrual cycle [5].

Major socio-emotional, biological and psychological changes that occur during puberty have the potential to alter developmental trajectories in the early years of adolescence [6, 7]. Previous research has explored several risk factors for menstrual irregularities, including age at menarche, moderate or vigorous exercise [8, 9] and obesity [10, 11]. In addition to the physical factors, work-related and psychosocial stress and mental illnesses were also reported as risk factors for menstrual disorders [12–16]. Epidemiological studies have shown that rates of depression are approximately equal between boys and girls before the onset of puberty, after which, rates for depression in girls are approximately twice that of boys [17, 18]. In this regard, increased mental health problems in adolescent girls during their early ages may cause neuroendocrine disruption, and chronic activation of the hypothalamic pituitary adrenal (HPA) axis during puberty that may lead to several types of menstrual irregularities [19].

Since most of the irreguarities associated with menstruation are due to the immaturity of the HPA axis, such disorders in adolescents are usually self-limiting [20]. There is growing evidence of the association of mental health problems including stress, anxiety, guilt, loneliness, dissatisfaction with life, suicidal thoughts and substance use in adolescent girls with their menstrual cycle dysfunction [12, 13, 21, 22]. A study on menstrual irregularities among medical students who had greater psychosocial stress showed that increased stress was significantly associated with occurrence of dysmenorrhoea and premenstrual symptoms that are severe enough to take medication [15].

Nevertheless, the problems related to menstruation are often ignored by primary health care and are generally perceived as minor health concern in most of the developing countries including India [23]. Identification of factors associated with abnormal menstrual patterns during adolescence may help prevent them in the earlier stage and enhance the reproductive health of adolescents and improve their quality of life. Therefore, the current study examines the prevalence of self-reported menstrual irregularities in adolescents using a large survey data and explores the association of depressive symptoms with menstrual irregularities in adolescents in two major states of Uttar Pradesh and Bihar in India. The study hypothesised that the depressive symptoms during adolescence are significantly associated with menstrual irregularities.

## Materials and methods

### Data

This study is based on the data obtained from the first round of the “Understanding the lives of adolescents and young adults" (UDAYA) survey. The survey was conducted in two states of India, namely, Bihar and Uttar Pradesh, in 2016 by the Population Council under the stewardship of the Ministry of Health and Family Welfare (MoHFW), Government of India. The survey aimed to explore the situation of younger (10–14 years) and older adolescent (15–19 years) girls and boys and the factors that influence the quality of transition from adolescence to young adulthood. The survey collected detailed information on education, economic activity, household work, migration, mass media exposure, social media exposure, aspirations, agency, gender role attitudes, awareness of sexual and reproductive health, romantic and sexual relationships, and health-seeking behavior. The UDAYA survey was designed to provide estimates for the state as a whole as well as for the urban and rural areas of the state for five categories of respondents. A total of 150 primary sampling units (PSUs) i.e. village in rural areas and census wards in urban areas have been selected in the state to achieve the required number of samples for interview. Further, the 150 PSUs were categorized equally into rural and urban areas, that is 75 for rural respondents and 75 for urban respondents. Within each sampling domain, a multi-stage systematic sampling design was used to select a representative sample. The 2011 census list of villages and wards (each consisting of several census enumeration blocks (CEBs) of 100–200 households) adopted as the sampling frame for the selection of village and wards in rural and urban areas, respectively. The list was stratified using four variables, namely, region, village/ward size, proportion to the population belonging to schedule castes and scheduled tribes and female literacy. The survey followed a three-stage sampling design in rural areas and four-stage in urban areas. Villages were first selected systematically from the stratified list as mentioned above, with selection probability proportional to size (PPS) in rural areas. In urban areas, 75 wards were first selected systematically with probability proportional to size (PPS) within each selected wards, then CEBs were arranged by administrative number and one CEB was randomly selected. Several CEBs adjacent to the selected CEB were merged to ensure at least 500 households for listing.The total sample consisted for Uttar Pradesh and Bihar was 10,350 and 10,350 adolescents, respectively. The required sample for each sub-group of adolescents from each state was determined at 920 younger boys (10–14 years), 2,350 older boys (15–19 years), 630 younger girls (10–14 years), 3,750 unmarried older girls (15–19 years), and 2,700 married older girls (15–19 years) in the state. Biomarkers were collected from all younger adolescents and a sub-sample of older adolescents. Details of sampling and data were mentioned in the survey report [24]. For this study, we excluded the data of 5,969 male respondents and 1,093 female respondents who had not yet experienced menarche and those who were currently pregnant (*n* = 825) at the time of the survey. Thus, we analyzed the data of 12,707 females aged 10–19 years in the present study.

### Variable description

#### Outcome variable

Menstrual irregularity was assessed through a direct question on self-reported menstrual problem in the survey: “Have you had menstrual problem in the last three months?”. Individuals who responded “no” were categorized as no menstrual irregularity, while those who answered “yes” were classified as having menstrual irregularity.

#### Covariates

The study includes various covariates that have potential influence on menstrual irregularity. The depressive symptoms of adolescent girls were determined through nine questions included in the questionnaire based on the Patient Health Questionnaire (PHQ-9). Individuals were asked these questions for the past two weeks or last 15 days of the time of the survey. These questions included, (a) had trouble falling asleep or sleeping too much, (b) feeling tired or having little energy (c) had poor appetite or overeating (d) had trouble in concentrating things (e) had little interest or pleasure in doing things (f) feeling down, hopeless or depressed (g) feeling bad about yourself (h) moving or speaking slowly or being restless (i) thought of hurting yourself or being dead off. All the questions were recorded on a scale of four, i.e., 0 as “not at all”, 1 as “less than once a week”, 2 as “one week or more”, and 3 as “nearly every day”. Further, a 27 point scale was prepared with cut-off points of 5, 10,15 and 20 that represent the thresholds for mild, moderate, moderate severely and severe depression, respectively. Then, depressive symptoms were categorized in four parts: 0 as “minimal”, 1 as “mild” 2 as “moderate” and 3 as “severe” [24].

Physical activity was categorized as “no” which involves who did not engage in physical activity and “yes” if respondents participated in walking, skipping, game or sports, running, yoga, etc. Substance use was classified as “no” and “yes”, based on response to self-reports of any form of tobacco, alcohol and drugs. Body mass index (BMI) was calculated by dividing the body weight (kg) by the square of the height (m^2^). Further BMI was categorized into three groups, i.e. underweight (- < 2sd) normal weight (l >  = 1sd & <  = 1sd) and overweight/obese (> 1sd) [24]. Girls use different items during menstrual period to prevent bloodstains from becoming evident which was determined as menstrual hygiene practices and recoded into three categories in the current study i.e. only sanitary napkin, only piece of cloth and both or other materials. Respondents were asked if they had ever experienced childbirth and the responses were dichotomized into no and yes. Other socio-demographic covariates included age of the respondents (10–14 and 15–19 years), year of schooling (no, 1–7 years, 8–9 years and 10 years and above), marital status (never married and married), wealth quintile (poorest, poorer, middle, richer and richest), caste (SC/ST, OBC and non-SC/ST/OBC), religion (Hindu and non-Hindus), and place of residence (rural and urban).

### Statistical analysis

Descriptive analysis was performed to describe the characteristics of the study population. Further, bivariate analysis with chi-square test was conducted to determine the menstrual irregularity by predictor variables. Sampling weight was used to estimate the percentage distribution in univariate and bivariate analysis. Finally, multivariable logistic regression models were carried out to determine the unadjusted and adjusted association between menstrual irregularity, depressive symptoms and other explanatory variables. Variance inflation factor (VIF) was estimated to check the multicollinearity among the variables used in the study [25], and there was no evidence of multi-collinearity. Results are presented in the form of unadjusted odds ratio (OR) and adjusted odds ratio (AOR) with 95% confidence interval (CI). All the statistical analysis was performed using STATA-14.

## Results

Characteristics of the study population are presented in Table 1. 11.22% of adolescent girls reported menstrual irregularity. The prevalence of mild, moderate and severe depressive symptoms among adolescents were 11.40%, 2.89% and 1.69%, respectively. Regarding health risk behaviors, one-third of girls were engaged in some type of physical activity, and 2.43% of adolescent girls were substance users. A 8.65% of adolescent girls were underweight. 45.13% of girls used pieces of cloth during the menstrual period to prevent bloodstains from becoming evident, and 14.32% of the participants experienced childbirth in the past. Most of the respondents were from 15–19 years old, rural areas, and Hindu religion. Around 13% of girls were illiterate. One-third of respondents (34.99%) were currently married. Nearly half of the respondents (46.76%) belonged to the higher wealth quintile.

**Table 1:** Characteristics of the study population

Characteristics	Frequency	Percentage
**Menstrual irregularity**		
No	11,373	88.78
Yes	1,334	11.22
**Depressive symptoms**		
Minimal	10,693	84.02
Mild	1,412	11.40
Moderate	369	2.89
Severe	233	1.69
**Physical Activities**		
No	8,045	66.62
Yes	4,662	33.38
**Substances use**		
No	12,432	97.57
Yes	275	2.43
**BMI Status**		
Underweight	284	8.65
Normal weight	2,887	87.87
Overweight/Obese	127	3.48
**Menstrual hygiene practices**		
Only sanitary napkin	4,259	28.9
Only piece of clothes	5,073	45.13
Others	3,375	25.97
**Childbirth experience**		
No	10,636	85.68
Yes	2,071	14.32
**Age**		
10–14	639	4.56
15–19	12,068	95.44
**Year of schooling**		
No	1,781	12.99
1–7 years	2,693	21.91
8–9 years	3,673	29.54
10 and above	4,560	35.56
**Marital status**		
Never married	8,333	65.01
Married	4,374	34.99
**Wealth Index**		
Poorest	1,526	13.16
Poorer	1,939	18.26
Middle	2,577	21.83
Richer	3,384	24.72
Richest	3,281	22.04
**Caste**		
Non SC/ST/OBC	2,385	18.82
SC/ST	2,868	24.99
OBC	7,454	56.19
**Religion**		
Non-Hindus	2,965	21.5
Hindu	9,742	78.5
**Residence**		
Rural	7,066	83.83
Urban	5,641	16.17
**State**		
UP	6,149	48.39
Bihar	6,558	51.61
**Total**	**12,707**	**100**

*Note*: *OBC* Other backward class, *SC* Schedule caste, *ST* Schedule tribe

The prevalence of self-reported menstrual irregularity among adolescent girls by their background characteristics is presented in Table 2. The prevalence of menstrual irregularity was significantly higher among adolescent girls with moderate and severe depressive symptoms (22%). Substance users (15.53%) had a higher prevalence of menstrual irregularity. Menstrual irregularity was higher among adolescent girls those who used sanitary napkin during the menstrual period (14.43%), married girls (13.06%), and rural residents (11.72%).

**Table 2:** Socio-economic and demographic characteristics among girls with self-reproted menstrual irregularity (*N* = 12,707)

Characteristics	Frequency	Percentage	*p*-value
**Depressive symptoms**			< 0.001
Minimal	958	9.42	
Mild	247	20.23	
Moderate	76	21.66	
Severe	53	21.98	
**Physical Activities**			0.100
No	872	11.66	
Yes	462	10.34	
**Substances use**			0.044
No	1295	11.11	
Yes	39	15.53	
**BMI status**			0.314
Under weight	24	7.9	
Normal weight	329	11.86	
Over weight	15	17.94	
**Menstrual hygiene practices**			< 0.001
Only sanitary napkin	403	14.43	
Only piece of clothes	394	9.81	
Others	537	10.28	
**Child birth experience**			< 0.001
No	1183	11.83	
Yes	151	7.54	
**Age**			< 0.001
10–14	11	5.88	
15–19	1293	11	
**Year of schooling**			0.592
No	201	11.29	
1–7 years	277	11	
8–9 years	372	10.68	
10 and above	484	11.78	
**Marital status**			0.002
Never married	824	10.23	
Married	510	13.06	
**Wealth Index**			0.082
Poorest	171	11.57	
Poorer	220	11.98	
Middle	279	11.3	
Richer	361	11.59	
Richest	303	9.89	
**Caste**			0.004
Non SC/ST/OBC	277	14.04	
SC/ST	331	11.86	
OBC	726	9.99	
**Religion**			0.091
Non-Hindus	336	11.94	
Hindu	998	11.02	
**Residence**			< 0.001
Rural	825	11.72	
Urban	509	8.61	
**State**			0.040
UP	681	11.50	
Bihar	653	10.62	
**Total**	1,334	11.22	

*Note*: *SC/ST* Scheduled Caste/Scheduled Tribe, *OBC* Other backward class, *p < 0.05

Table 3 shows the unadjusted and adjusted odds ratio of self-reported menstrual irregularity by different background characteristics among adolescent girls. Model-2 was adjusted with physical activity, substance use, menstrual hygiene practices, and childbirth experience. Model-3 was adjusted with other socioeconomic, demographic and behavioural characteristics. Unadjusted result (model-1) shows that the probability of menstrual irregularity was significantly more than two times higher among adolescents having depressive symptoms. Adolescent girls with mild (OR: 2.15, CI: 1.85–2.51), moderate (OR: 2.64, CI: 2.03–3.42) and severe depressive symptoms (OR: 2.99, CI: 2.19–4.09) were significantly 2.15 times, 2.64 times and 2.99 times more likely to have menstrual irregularity as compared to those who had minimal depressive symptoms, respectively. The magnitude of menstrual irregularity has been slightly changed when estimates were adjusted for several covariates in model-3. Physically active adolescents girls were less likely (AOR: 0.82, CI: 0.73–0.93) than physically inactive girls. Adolescent girls who used piece of cloth (AOR: 1.17; CI: 1.02–1.35) and either napkin or cloth or other materials (AOR: 1.32; CI: 1.14–1.54) for menstrual hygiene practices had a higher likelihood of menstrual irregularity as compared to those who used only sanitary napkins. Adolescent girls who experienced childbirth showed lower risk of menstrual irregularity (AOR: 0.55; CI: 0.45–0.65) than their counterparts.

**Table 3:** Odds ratio of self-reported menstrual irregularity among adolescent girls by background characteristics (*N* = 12,707)

Covariates	Model-1	Model-2	Model-3
	**Unajusted Odds Ratio (95% CI)**	**Adjusted Odds Ratio (95% CI)**	**Adjusted Odds Ratio (95% CI)**
**Depressive symptoms**			
Minimal®			
Mild	2.15* (1.85–2.51)	2.22* (1.90–2.59)	2.16* (1.85–2.52)
Moderate	2.64* (2.03–3.42)	2.67* (2.05–3.47)	2.58* (1.98–3.37)
Severe	2.99* (2.19–4.09)	3.08* (2.25–4.23)	3.11* (2.26–4.27)
**Physical Activities**			
No®			
Yes		0.82* (0.73–0.93)	0.95 (0.84–1.09)
**Substances use**			
No®			
Yes		1.25 (0.88–1.77)	1.16 (0.82–1.66)
**Menstrual hygiene practices**			
Only sanitary napkin®			
Only piece of cloth		1.17* (1.02–1.35)	1.06 (0.91–1.25)
Others		1.32* (1.14–1.54)	1.23* (1.06–1.44)
**Childbirth experience**			
No®			
Yes		0.55* (0.45–0.65)	0.41* (0.34–0.51)
**Age (Years)**			
10–14®			
15–19			1.43* (1.02–2.00)
**Year of schooling**			
Iliterate®			
1–7 years			0.95 (0.78–1.17)
8–9 years			0.94 (0.77–1.15)
10 and above			1.04 (0.84–1.27)
**Marital status**			
Not married®			
Currently married			1.56* (1.34–1.80)
**Wealth Index**			
Poorest®			
Poorer			0.99 (0.79–1.23)
Middle			0.97 (0.79–1.20)
Richer			0.97 (0.79–1.20)
Richest			0.87 (0.69–1.11)
**Caste**			
Non SC/ST/OBC®			
SC/ST			0.93 (0.76–1.12)
OBC			0.77* (0.66–0.90)
**Religion**			
Non-Hindus®			
Hindu			0.83* (0.72–0.97)
**Residence**			
Rural®			
Urban			0.76* (0.67–0.87)
**State**			
UP®			
Bihar			0.94 (0.83–1.06)

*Note*: *SC/ST* Scheduled Caste/Scheduled Tribe, *OBC* Other backward class, *CI* Condidence interval, *p < 0.05, ® Reference category

After adjusting for other sociodemographic and economic covariates in model-3, the association between menstrual irregularity and depressive symptoms remained significant; however, we did not find any significant association between menstrual irregularity and physical activity, substance use, year of schooling and wealth quintile. The menstrual irregularity was significantly higher in adolescents aged 15–19 years (AOR: 1.43; 95% CI: 1.020–2.002) and married (AOR: 1.56; CI: 1.342–1.804) than in girls aged 10–14 years and unmarried respectively. OBCs and Hindu religion were found to be negatively associated with menstrual irregularity than non-SC/ST/OBC and non-Hindus, respectively. Adolescent girls from urban areas were less likely (AOR: 0.76; CI: 0.668–0.869) to have menstrual irregularity than their rural counterparts.

## Discussion

Irregular menstruation is a significant indicator of present and prospective health issues in women. It is a common problem among adolescent girls and has an impact on their day-to-day activities. However, it receives little attention, particularly in developing and underdeveloped nations. The present study was conducted to explore the prevalence of self-reported menstrual irregularity among adolescent girls and to investigate the association of depressive symptoms with irregular menstruation. In this population-based study of Indian adolescents, 11.2 percent of girls aged 10–19 years reported having irregular menstruation, which was lower than previously reported in various parts of India [26–30]. A study in the northern part of India has reported that nearly 28.7 percent of adolescent girls experienced irregular menstruation [28]. According to studies from southern India, 23–38 percent of college students have menstrual irregularities [26, 29, 31]. Some of the reasons for this variation include disparities in the definition of irregular menstruation employed by researchers and the age distribution of participants. In addition, during a face-to-face interview, individuals may be hesitant to discuss menstrual problems.

There exists a bidirectional relationship between depression and the menstrual problems. The menstrual cycle and mental health are interlinked, and anomalies in one can have an impact on the other [32]. Hormonal changes in the body predispose women to depression. On the other hand, depression alters the hypothalamic-pituitary axis (HPA), which leads to menstrual irregularities [33]. In the present study, a significant positive association was observed between depressive symptoms and menstrual irregularity among adolescent girls. In particular, individuals with moderate and severe depressive symptoms were 2.6 and 2.9 times more likely to report menstrual irregularity. This association remained significant even after adjusting for several confounding factors like physical activity, substance use, menstrual hygiene practices, childbirth experience, age, years of schooling, marital status, wealth index, caste, religion, place of residence etc. Furthermore, the likelihood of menstrual irregularity tends to rise with the increase in the level of depressive symptoms. The result is consistent with the findings of previous research [34, 35]. A recent study conducted among undergraduate female students in Ethiopia also found a significant association of perceived stress with menstrual irregularity [36]. Jung et al. (2017), in their study on irregular menstrual cycles in Korean women, have shown that women with perceived stress and depressive moods had 1.46 and 2.07 times higher odds of irregular menstruation, respectively [37].

One theory that explains the mechanism through which depressive mood can affect the menstrual cycle involves the HPA axis. Stress has been linked to neuroendocrine disturbance in children, with the HPA axis being particularly affected [38]. As a result, females who are under a lot of stress or have a depressed mood may have more irregular menstrual periods than those who aren't [39, 40]. Physical and physiological stress have a high impact on the female reproductive system. Although modest amounts of stress can encourage people to adapt to changes, take on challenges, and accomplish tasks, extreme levels of stress can be harmful to one's physical and mental health. Menstrual function can be disturbed if the corticotrophin-releasing hormone system is triggered in response to stress [19]. However, further studies with longitudinal design should be implemented to determine the causative relationship between depression and menstrual irregularities among adolescents.

Several studies have documented a significant association of smoking status and alcohol consumption with irregular menstrual cycles among women [36, 41, 42]. Literature suggests that smoking or alcohol consumption elevates the levels of estrogen, testosterone, and luteinizing hormone in women [43, 44], which may result in irregular menstruation. Similarly, multiple studies in India found that lack of physical activity and higher levels of junk food are associated with menstrual problems among adolescents [28, 45, 46]. In the present study, girls who did no physical activity, smoked, drank alcohol, or used other substances had significantly higher chances of reporting irregular menstrual cycles as compared to those who did physical activity or never use such substances, respectively. However, the significance disappeared in the multivariable analysis. The findings are in agreement with a study conducted in Korea which found no significant association of alcohol consumption and regular exercise with menstrual irregularities [47]. The discrepancy between earlier and current research on lifestyle factors in association with menstrual problems might be attributed to the diversity of characteristics of the study participants and factors included in the analysis. Thus, insignificant results of physical activity and substance use in the fully adjusted multivariable model need to be further investigated. Our study also revealed that the use of unhygienic menstrual absorbents such as old clothes or other materials was association with a greater likelihood of experiencing menstrual irregularity. Unhygienic menstrual practices can also lead to several serious and long-term health implications like gastrointestinal, vaginal, and perineal infections, recurring reproductive tract infections (RTIs) and even cervical cancer [48–50]. In accordance with the findings of a study by Kwak et al. (2019) [4], we observed a significant difference in the prevalence of menstrual irregularity among girls according to childbirth status.

It is evident from the current study that irregular menstrual cycles were significantly more common among older adolescents (15–19 years) as compared to the younger adolescents group. This result is inconsistent with the findings of previous literature that has documented increasing age as the protective factor for irregular menstruation [47, 51]. However, Hahn et al. (2013) have found that increasing age is associated with higher odds of menstrual irregularity [42]. The psychological burden of good academic performance in 10th or 12th grades as well as the college entrance exam might be one possible reason for the high prevalence of irregular menstruation among adolescents in the age group of 15–19 years. Further, the analyses showed that girls residing in urban areas were less likely to report menstrual irregularity than rural girls.

There are numerous limitations to our study that should be considered while interpreting the results. Firstly, we cannot infer the direction of causal relationship between the factors and irregular menstruation due to the cross-sectional design of the study. Secondly, menstrual irregularity was assessed exclusively on the basis of self-reports, which can be subjected to reporting bias. Third, since the study was based on the secondary analysis of the data, we could not include all the factors known to be related to irregular menstruation. As a result, uncontrolled confounding of our findings is possible, restricting the interpretation of the data even more. Future studies are warranted using longitudinal/cohort design and including more information on types and duration of irregularity and other confounding variables such as diatery pattern, sleep quality and physical illnesses which may have significant association with both menstrual problems and depression.

## Conclusion

A sizable proportion of adolescent girls in the study sample had irregular menstruation. Also, a significant association of depressive symptoms with self-reported menstrual irregularity among adolescent girls was observed. Therefore, while treating females with irregular menstrual cycles, clinicians may need to pay greater attention to their mental health problems. In addition, the study also suggests that hygienic menstrual practices such as the use of sanitary napkins, childbirth experience, and urban residency seem to be protective factors against menstrual irregularity. Given the association between menstrual hygiene practices and menstrual irregularity, improvement in hygiene practice behaviour should be emphasized.

## Data Availability

The study utilizes a secondary source of data that is freely available in the public domain through: https://dataverse.harvard.edu/dataset.xhtml?persistentId=doi:10.7910/DVN/RRXQNT.
